# Molecular Evolution of SARS-CoV-2 during the COVID-19 Pandemic

**DOI:** 10.3390/genes14020407

**Published:** 2023-02-04

**Authors:** Luis Daniel González-Vázquez, Miguel Arenas

**Affiliations:** 1 Biomedical Research Center (CINBIO), University of Vigo, 36310 Vigo, Spain; 2 Department of Biochemistry, Genetics and Immunology, University of Vigo, 36310 Vigo, Spain; 3 Galicia Sur Health Research Institute (IIS Galicia Sur), 36310 Vigo, Spain

**Keywords:** molecular evolution, SARS-CoV-2, recombination, molecular adaptation, rate of evolution, genetic diversity

## Abstract

The severe acute respiratory syndrome coronavirus 2 (SARS-CoV-2) produced diverse molecular variants during its recent expansion in humans that caused different transmissibility and severity of the associated disease as well as resistance to monoclonal antibodies and polyclonal sera, among other treatments. In order to understand the causes and consequences of the observed SARS-CoV-2 molecular diversity, a variety of recent studies investigated the molecular evolution of this virus during its expansion in humans. In general, this virus evolves with a moderate rate of evolution, in the order of 10^−3^–10^−4^ substitutions per site and per year, which presents continuous fluctuations over time. Despite its origin being frequently associated with recombination events between related coronaviruses, little evidence of recombination was detected, and it was mostly located in the spike coding region. Molecular adaptation is heterogeneous among SARS-CoV-2 genes. Although most of the genes evolved under purifying selection, several genes showed genetic signatures of diversifying selection, including a number of positively selected sites that affect proteins relevant for the virus replication. Here, we review current knowledge about the molecular evolution of SARS-CoV-2 in humans, including the emergence and establishment of variants of concern. We also clarify relationships between the nomenclatures of SARS-CoV-2 lineages. We conclude that the molecular evolution of this virus should be monitored over time for predicting relevant phenotypic consequences and designing future efficient treatments.

## 1. Introduction

After the severe acute respiratory syndrome coronavirus (SARS-CoV) identified in China in 2002 [1] and the Middle East respiratory syndrome coronavirus (MERS-CoV) detected in 2012 in Saudi Arabia [2], the SARS-CoV-2 caused another severe respiratory disease in humans. The expansion of SARS-CoV-2 to humans started in late 2019 in Wuhan, China [3], and caused a worldwide pandemic with innumerable economic, social, and political consequences [4]. This virus was also transmitted from humans to other animals, such as mice, hamsters, cats, dogs, ferrets, and minks, among others [5,6]. Similarly to SARS-CoV, SARS-CoV-2 is a member of the Orthocoronavirinae subfamily, subgenus Sarbecovirus, and presents a positive sense single-stranded RNA genome with a length of approximately 29.9 kb [7,8]. During the pandemic, the genome of SARS-CoV-2 evolved, producing variants that displayed different infective and immunological properties [9,10], which caused a series of epidemiological waves around the world. Clearly, understanding the molecular evolution of SARS-CoV-2 is a key factor in predicting the future of the pandemic and designing more durable treatments, including vaccines and antiviral drugs [11,12,13]. Indeed, since SARS-CoV-2 is a newly emerging virus that infects humans with serious consequences, understanding its molecular adaptation to our species and treatments is required for public health [14].

## 2. The SARS-CoV-2 Genome and Proteins

**Figure 1:**
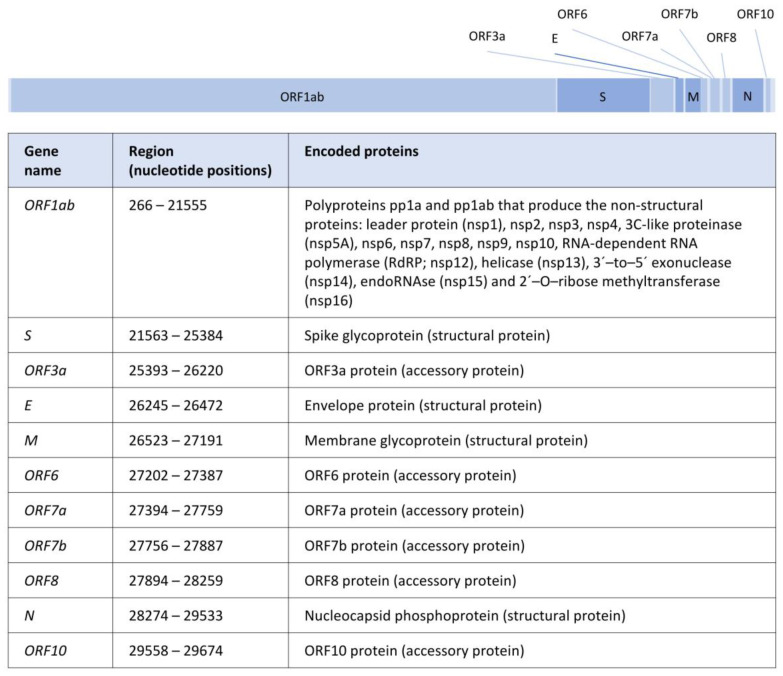
Illustration of the SARS-CoV-2 genome structure. Scaled representation of genes along the SARS-CoV-2 genome, including a table with the nucleotide positions between each ORF (Open Reading Frame) and the corresponding encoded proteins according to https://www.ncbi.nlm.nih.gov/nuccore/1798174254 (accessed on 20 November 2022). Structural protein-coding genes are shown in dark blue.

The almost 30 kb of the SARS-CoV-2 genome contains 11 genes that encode for 29 proteins, including nonstructural, structural, and accessory proteins [8,15] (Figure 1). These proteins are involved in the host cell recognition, entry and uncoating, replication and transcription, assembly, and release, among other functions (details below). In particular, the four genes coding for structural proteins, from 5′ to 3′, are the gene *S* (nucleotide positions 21563–25384, encoding the spike glycoprotein [16]), the gene *E* (nucleotide positions 26245–26472, which produces the viral envelope proteins [17]), the gene *M* (nucleotide positions 26523–27191, leading to the membrane M protein [17]), and the gene *N* (nucleotide positions 28274–29533, encoding nucleocapsid N proteins [18]).

Briefly (see also Figure 1), the spike glycoprotein binds the virus to the cell receptor; thus, its diversity should be considered in studies on the transmissibility of the virus and the design of certain therapies [19]. Within this protein, the S1 subunit includes a receptor-binding domain called RBD that contacts with the human angiotensin-converting enzyme 2 (ACE2) [16]. The viral envelope proteins encoded by the gene *E* are involved in the assembly and release of virions, as well as in ion transport and induction of host cell apoptosis [15,20], and also are often conserved in nearby coronaviruses [21]. The membrane M protein performs RNA packaging in the viral assembly by interacting with the N protein (see later) [22], and it is also conserved among related coronaviruses [21]. The nucleocapsid N proteins also participate in RNA packaging, providing stability to the viral assembly and transcription [20,23]. In addition, these proteins can antagonize antiviral interfering RNA and, by inhibition of cyclin-CDK, they can change the cell to the S phase where DNA duplication occurs [15]. The non-structural protein RNA-dependent RNA polymerase (RdRP) participates in the viral replication using a strand of RNA (template) to synthesize the new strand [24].

## 3. The Nomenclature and Evolutionary History of SARS-CoV-2 Lineages

A variety of SARS-CoV-2 lineages emerged during the expansion of this virus in humans, and their nomenclatures differ among authors or entities, producing some confusion. The most used nomenclature of SARS-CoV-2 lineages is the PANGO nomenclature [25]. It consists of two initial lineages (named with a letter, A and B) that produced sublineages represented by adding numerical sublevels (i.e., B.1 and B.1.6). Next, when the sublineages exceed three sublevels, a new letter is used instead of adding a fourth numerical sublevel (i.e., B.1.1.28.2 is named P.2 and B.1.1.7.7 is named Q.7) [26]. Another nomenclature was presented by Nextstrain [27], a project that phylogenetically classifies SARS-CoV-2 genomes available from the GISAID database [28]. The nomenclature of Nextstrain is based on the different clades that were detected over time. A third nomenclature was presented by GISAID. This nomenclature is based on letters assigned to molecular markers of clades of interest (i.e., the marker S-D614G was defined as clade G, and the subsequent marker S-A222V arising in clade G was defined as clade GV). These clades often display a direct association with PANGO lineages (i.e., GISAID GR corresponds to Nextstrain 20B and PANGO B.1.1.*). Next, WHO (World Health Organization) presented an additional classification of SARS-CoV-2 lineages and clades according to their relevance for the pandemic. In particular, WHO used the terms variant of concern (VOC), a variant of interest (VOI), and a variant under monitoring (VUM). This nomenclature of lineages and clades uses Greek letters (i.e., Alpha and Beta variants), and the number of considered variants increased with the real-time monitoring of the pandemic. A correspondence between PANGO, Nextstrain, GISAID, and WHO nomenclatures can be found in [26].

**Figure 2:**
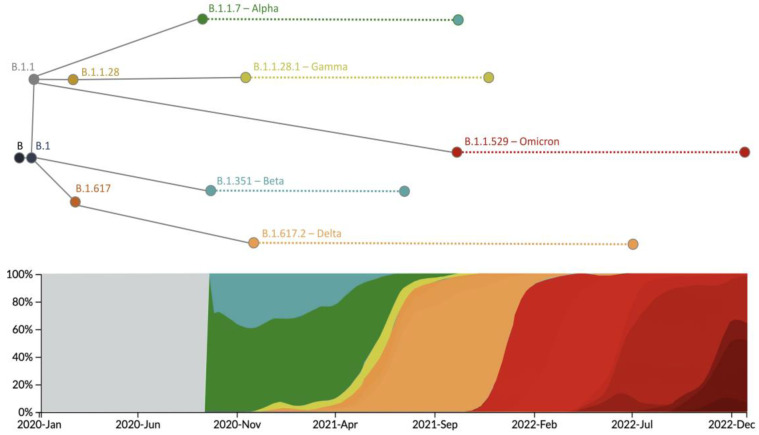
Evolutionary history of main SARS-CoV-2 VOCs. This illustration shows the emergence, establishment, and genetic relationships of the main VOCs according to current knowledge. It also includes a graph describing the predominance of every VOC over time, which we obtained with Nextstrain based on data from GISAID [27,28].

The SARS-CoV-2 genome was highly sequenced, providing a clear view of the evolutionary history of the main lineages of the virus, especially the VOC lineages (Alpha, Beta, Gamma, Delta, and Omicron). In Table 1 and Table 2, we show the mutations that define each VOC lineage, and in Figure 2, we illustrate their frequency over time and their main phylogenetic relationships. In 2020, the first VOC, the Alpha lineage (B.1.1.7), emerged after the accumulation of several genetic changes in the gene *S* [29,30] (Table 1), being responsible for an increase in transmissibility between 40% and 90%, respect to previous lineages [29,31]. Subsequently, the Beta lineage (B.1.351) appeared [32] with several mutations also in the gene *S* (Table 1), one of them (E484K) shared with the Alpha lineage. This genetic variation increased the affinity between the spike protein and the human ACE2 receptor with a subsequent increase in transmissibility up to 50% compared to previous lineages [31]. The lineage B.1.1.28 produced a third VOC, the Gamma variant (P.1) [31,33], which presented several genetic changes in the gene *S* (Table 1), some of them (N501Y, K417N, and E484K) shared with other previous VOCs, and that increased the affinity with ACE2 and, thus, the transmissibility [31]. In addition, Sabino et al. [34] indicated that this variant presents a higher rate of reinfection compared to previous variants. At the end of 2020, the Delta variant (B.1.617.2) emerged (Figure 2), displaying many additional mutations (Table 1) that produced an increase in transmissibility (higher binding stability with ACE2 [31]) and a rapid spread throughout the world, with greater severity and causing more ICU admissions and deaths [35]. In 2021 the Omicron variant (B.1.1.529 or BA) was detected [36,37]. It presented many mutations in the gene *S* (Table 2), especially in the coding region RBD, and some of them were shared with previous variants (K417N, T478K, E484K, N501Y, and D614G). A crucial aspect of this variant is its capacity to reduce immunity in vaccinated populations [32,38]. When Omicron becomes the dominant variant, its lineages, BA.1, BA.2, BA.3, BA.4, BA.5, and their descendants (i.e., BA.1.1, BA.2.12.1, BA.2.11, BA.2.75, and BA.4.6), emerged and increased in frequency. These lineages are derived from the accumulation of diverse mutations (Table 2) that increased infectivity and immune escape (i.e., BA.4 and BA.5 showed infectivity higher than BA.2, which, in turn, displayed higher infectivity than BA.1) [39]. Interestingly, multiple mutations were fixed in parallel in different VOCs through adaptation, considering their potential factoring in diverse major viral traits (Table 1 and Table 2, specific examples are illustrated in Table 3). For additional information about VOCs, the reader is referred to the reviews [31,36].

## 4. Evolutionary Mechanisms of SARS-CoV-2

### 4.1. The Mutation Process in SARS-CoV-2

A variety of viruses present high mutation rates, which, coupled with large population sizes, can result in a large genetic variability. Note that increasing genetic diversity is a key feature for the survival and pathogenesis of RNA viruses [57]. Thus, RNA viruses often present highly error-prone RNA polymerases that cause multiple mutations, but so far, SARS-CoV-2 has shown a moderate acquisition of mutations [58,59,60,61,62]. In particular, coronaviruses present a global mutation rate of around 10^−6^ per base and per infection cycle [61] (10^−3^ per site and year [62]), which varies among genes and where the highest rate in SARS-CoV-2 was detected in the *S* gene [63]. Mutations that occur in the viral surface of proteins are extremely important for generating antigenic variants that allow the virus to evade host immune surveillance, and they can also affect epidemiological and pathogenic characteristics, such as the basic reproductive number (R0), transmissibility, and mortality [64,65]. Indeed, certain mutations allow the virus to escape from the activity of antiviral treatments [66,67]. Interestingly, coevolving sites were detected in key SARS-CoV-2 proteins, such as the spike protein, probably as a consequence of protein stability, affinity, and interaction patterns under specific within-host pressures [43,68]. SARS-CoV-2 is a relatively recent virus infecting humans; thus, we believe that its genetic diversity will largely increase over time.

### 4.2. Recombination in the SARS-CoV-2

Recombination is a fundamental process in the evolution of multiple viruses and can produce new variants, better adapted to the immune system of the host and antiviral therapies [69]. Moreover, the identification of recombination is crucial to avoid biases in diverse phylogenetic analyses, such as phylogenetic tree reconstruction [70], ancestral sequence reconstruction [71], and detection of selection [72]. In RNA viruses, recombination can occur in the simultaneous infection (by two or more viruses) of the same host cell to produce a redistribution of mutations in the resulting recombinant genome [73]. Next, natural selection can operate upon these new genetic variants [74,75]. The recombination rate in the family Coronaviridae is relatively high [73]. However, this was not observed in the SARS-CoV-2, despite this coronavirus probably originating by recombination between other coronaviruses [76,77]. Multiple studies investigated the evidence of recombination along the genome of SARS-CoV-2, and most of them found that recombination events are scarce, perhaps because of a low recombination rate or because the overall low genetic diversity present in the virus made the detection of recombination difficult [78,79,80]. Thus, despite the fact that several studies found a lack of recombination [81,82], others could detect some evidence (details shown in Table 4). Concerning the latter, the detected recombination breakpoints mainly involved the genes *ORF1ab* and *S* [83,84]. Next, to our knowledge, in contrast with other RNA viruses [85,86,87], the population recombination rate in SARS-CoV-2 was not yet investigated. A key factor in obtaining clearer estimates of recombination is to overcome the huge computational burden required to analyze millions of currently available genome sequences [80]. In general, recombination is an evolutionary force that could change the course of the evolution of this virus by producing variants with improved transmissibility, infectivity, and resistance to therapies; thus, we believe that it should be seriously taken into account.

### 4.3. The Rate of Molecular Evolution of the SARS-CoV-2

The overall rate of molecular evolution of SARS-CoV-2 ranges from 10^−3^ to 10^−4^ [81,110,111,112,113,114,115,116,117] (Table 5). However, different rates of evolution were identified among VOCs. In particular, the variant Alpha presented a rate of evolution of 8.47 × 10^−3^ (0.49–0.62 × 10^−3^), the variant Beta showed 1.71 × 10^−3^ (0.34–33.20 × 10^−3^), the variant Gamma showed 2.76 × 10^−3^ (1.21–13.23 × 10^−3^), and the variant Delta presented 1.54 × 10^−3^ (0.62–7.35 × 10^−3^) in substitutions per site and year (with 95% confidence interval) [118]. These rates were higher than those detected for non-VOC, which presented estimates of 0.53 × 10^−3^ (0.49–0.62 × 10^−3^) substitutions per site and year [118]. The rate of evolution in SARS-CoV-2 was also studied over time to evaluate the hypothesis of the molecular clock (constant rate of evolution over time [119]). Several authors [81,110,116] found that a relaxed molecular clock model fits better with the SARS-CoV-2 genome evolution when compared to a strict molecular clock model, suggesting that the rate of evolution changed over time. In this concern, Tay et al. [118] observed that the evolution of data collected at the beginning of the pandemic was better explained by a strict molecular clock model, while a relaxed molecular clock model could better fit with data collected more recently.

### 4.4. Molecular Adaptation in the SARS-CoV-2 Genome

The selection was widely investigated in SARS-CoV-2, especially using the traditional metric nonsynonymous/synonymous substitution rate ratio (dN/dS) [120]. At the global (whole genome) level, genetic signatures of negative (purifying) selection were observed, suggesting overall maintenance of the function of protein variants [62,121,122,123]. At the gene level, the negative selection was detected in most of the SARS-CoV-2 genes, including *S* [124,125,126,127,128], *E* [124,125,126,128], *M* [124,125,128], *N* [124,125,126,128], *ORF6* [124,125,128], *ORF7a* [124,125], *ORF7b* [124], *ORF8* [124,128], *ORF10* [124], and also in regions, such as *ORF1ab* coding for nonstructural proteins [124,125,126,127,128]. By contrast, the positive (diversifying) selection was detected in several genes, including *S* [121,129], *N* [121,129], *ORF3a* [121,129], *ORF8* [121,129], and in the coding regions of some non-structural proteins, such as nsp4 [121], nsp5A [128], nsp10 [128], and nsp13 [121]. Interestingly, some studies identified positive selection at the whole gene level in genes, for which other studies observed negative selection, for example, in *ORF7a* and *ORF10* [128], suggesting that further analyses should be performed to clarify these findings. We highlight the positive selection detected in the gene *S*, especially in sites of the RBD, since those nonsynonymous changes can affect the binding of the spike protein with the ACE2 receptor (details below) [130]. Next, a number of studies detected positively selected sites (PSSs) in the genes *S* [20,122,123,131], *N* [20,122,123], *ORF3a* [123,126], *ORF8* [123,126], and in the coding regions of the non-structural proteins nsp2 [129], nsp3 [129], nsp4 [129], nsp6 [123,129], nsp12 [129], and nsp13 [123,129]. Some of them were relevant, such as the PSS, associated with the D614G mutation in the gene *S* [108,122], which participates in the opening of the RBD [20] and produces an advantage for infectivity [48] and transmissibility [132]. In the same gene, a PSS associated with the L5F mutation facilitated the folding, assembly, and secretion of the virus [20], and PSSs associated with the mutations K417T, E484K, and L18F (also the N501Y mutation [133,134]) allowed escape from monoclonal antibodies [135,136,137,138]. Indeed, other detected PSSs were associated with the P681H mutation that increased the cleavage of spike protein with ACE2 [139] and the I82T mutation that increased the stability of the protein M [140]. In the gene *ORF3a*, a PSS associated with the Q57H mutation increased the affinity between the proteins ORF3a, spike, and M, favoring viral replication [141,142]. In the protein nsp6, a relevant PSS involved the L37F mutation that allowed the virus to reduce the cellular defense via autophagy regulation [42]. Concerning the region coding for nsp12 and nsp14 (RdRP), some PSSs were detected and related with the mutations P323 (nsp12), A394V, and I42V (nsp14) that were associated with promoting an increase in the mutation rate [121]. Of course, many negatively selected sites (NSSs) and coevolving sites were also identified along the genome and were associated with fundamental processes that should be maintained for viral replication [143].

#### Molecular Adaptation Induced by Therapies and Immune Systems

Genetic signatures of molecular adaptation in SARS-CoV-2 can also be observed as a consequence of the applied therapies and the immune systems. Concerning the latter, in the presence of the virus, the immune system operates through different mechanisms, such as CD8+ T and natural killer (NK) cells. Indeed, subepithelial dendritic cells and macrophages induce the differentiation of CD4+ T cells into memory T helper types Th1, Th17, and follicular T helper. The latter helps B cells to become plasma cells, promoting the production of IgM, IgA, and IgG antibodies [144].

Actually, the fixation of escape variants in virus populations was already observed [145,146], where multiple variants were able to evade therapeutic antibodies through escape mutations [145,146,147]. For example, the Beta and Gamma variants presented the mutation N439K that considerably increased the neutralizing activity of monoclonal antibodies and polyclonal serum [131,145,147]. Indeed, these variants often included the mutation K417N which also favors viral escape from diverse monoclonal antibodies, and the mutation N501Y (also observed in the Alpha and Omicron variants), which increases transmissibility through a higher affinity with ACE2 [147]. Therefore, therapeutic monoclonal antibodies, such as Casirivimab and Imdevimab, showed a smaller efficacy due to mutations, such as K417N and E484K (present in the Beta variant) for the former antibody, and mutations, such as L452R/Q (present in the Beta and Delta variants) for the latter [137,148]. Indeed, certain mutations observed in the Gamma and Omicron variants also reduced the efficacy of therapeutic monoclonal antibodies, such as Bamlanivimab (used to treat infection with the Gamma variant) [149] and Bamlanivimab, Etesevimab, Casirivimab, Imdevimab, and Regdanvimab (used against the Omicron variant) [67]. Moreover, the selection was not only detected to fix variants that escape from the recognition of monoclonal antibodies but also to fix the variants resistant to polyclonal serum and plasma. For example, Greaney et al. [147] identified mutations in the RBD region of the spike protein that reduced the efficacy of both types of antibodies.

Concerning vaccines, they produced strong selective pressures that caused the fixation of previously neutral or non-beneficial genetic changes. For example, some authors reported that the Novavax vaccine, which displayed an efficacy of 95.6% against the original SARS-CoV-2 variant, only presented an efficacy of 85.6% against the Alpha variant and 60% against the Beta variant [150]. Indeed, several studies showed that some mutations (i.e., E484K in the Beta variant and L452R in the Delta variant) caused resistance to vaccines based on mRNA (i.e., Pfizer and Moderna) and adenoviral vectors (i.e., Johnson & Johnson) [137,149,151]. In addition, the Gamma variant displayed resistance in Pfizer-vaccinated patients [149], and the Omicron variant showed escape in patients vaccinated with Pfizer, AstraZeneca, and Moderna [67,152]. Next, some studies detected mutations associated with resistance to Remdesivir, the most widespread antiviral treatment against SARS-CoV-2. These mutations in the gene *S*, which circulated at low frequency, were A97V [153], F480L/S/C [154], V557L [154], and E802D [155,156]. These findings suggest the need for continuous monitoring of SARS-CoV-2 molecular evolution for designing effective therapies against the variants circulating at every time. Indeed and despite the cited resistant mutants, vaccination largely reduces illness, hospitalization, and mortality [157,158]. In this concern, efforts should be made to provide access to vaccines in low-income countries, and there is a general need to improve the equitability of vaccination coverage worldwide [159].

## 5. Conclusions and Future Prospects

The SARS-CoV-2 pandemic promoted an impressive and convenient amount of works about this virus. Note that so far, more than 14 million SARS-CoV-2 genomes have been deposited in GISAID. This large amount of data allowed us to properly study the molecular evolution of this virus, identify the relationships between phenotypic (i.e., transmissibility and severity of the disease) and molecular (i.e., mutations) observations, and even predict which therapies are more appropriate at every time (for every variant). Samples of SARS-CoV-2 collected in humans overall presented genetic signatures of moderate mutation rate, little recombination, and diverse selective pressures (including positively selected sites, where some of them were promoted by treatments). Interestingly, several studies indicated that these evolutionary patterns could be changing over time, in particular toward increasing the rate of evolution (including a higher frequency of recombination events) and, consequently, the emergence and fixation of new variants. This trend could also be favored by the extremely large virus populations and the capacity of RNA viruses to adapt to new environments, such as those imposed by immune systems and therapies.

The pandemic of SARS-CoV-2 is far from over, and the virus continues circulating and evolving with sufficient capacity to produce variants presenting antiviral resistance. Therefore, monitoring SARS-CoV-2 evolution is extremely useful for designing treatments effective at each period of time. Moreover, we believe that efforts should be made to obtain a precise fitness landscape that includes the observed evolutionary trajectories of the virus, considering both mutation and recombination events, to better understand the relationships between those molecular changes and the observed phenotypic consequences. We also believe that in addition to the large amount of genomic data that is currently available for this virus, and that continues to increase, efforts should also be made to develop computational frameworks for the evolutionary analyses of such a large amount of data (see [160]). Future zoonoses events involving coronaviruses seem inevitable [11] and, in this concern, the knowledge that we can learn from the molecular evolution of SARS-CoV-2 could be useful to improve the prevention, anticipation, and management of future pandemics caused by similar viruses.

## Figures and Tables

**Table 1:** Mutations for each variant of concern presented by WHO to date (excluding Omicron, shown in Table 2). The presented main amino acid (Aa) and deletion (Del) mutations include their position in the corresponding protein or the entire genome, respectively [40]. Mutations present in different VOCs (including Omicron, Table 2), which constitute parallel mutations, are shown in italics.

VOC (PANGO Lineage)	Detected Mutations that Define VOCs (Except Omicron, Table 2) at Each Gene										
	*ORF1a*	*ORF1b*	*S*	*ORF3a*	*E*	*M*	*ORF6*	*ORF7a*	*ORF7b*	*ORF8*	*N*
Alpha (B.1.1.7)	Aa: T1001I A1708D I2230T Del: *3675–3677*	Aa: *P314L*	Aa: P9L V483F *E484K* F486I *Q498R N501Y* A570D *D614G P681H* T716I S982A D1118H Del: H69 V70							Aa: Q27* R52I Y73C *S84L*	Aa: D3L *R203K G204R* S235F
Beta (B.1.351)	Aa: T265I K1655N K3353R Del: *3675–3677*	Aa: *P314L*	Aa: S13I D80A D215G, *K417N E484K N501Y D614G* A701V A879S Del: L242 A243 L244	Aa: Q57H S171L	Aa: P71L					Aa: *S84L*	Aa: T205I
Gamma (P.1)	Aa: S1188L K1795Q Del: *3675–3677*	Aa: *P314L* E1264D	Aa: L18F T20N P26S R78M D138Y R190S K417T *E484K N501Y D614G H655Y* T1027I V1176F	Aa: S253P						Aa: *S84L* E92K	Aa: P80R *R203K G204R*
Delta (B.617.2)		Aa: *P314L* G662S P1000L	Aa: T19R *T95I G142D* E156G *L452R T478K* R567I *D614G H655Y* P681R D950N T1117I *Del*: F157 R158	Aa: S26L		Aa: I82T		Aa: V82A, T120I		Aa: *S84L* Del: 119–120	Aa: D63G R203M D377Y

**Table 2:** Mutations for each lineage of the Omicron variant of concern. The presented main amino acid (Aa), nucleotide (Nt), insertion (Ins), and deletion (Del) mutations include their position in the corresponding protein or the entire genome, respectively [39,40,41]. Mutations present in different VOCs (including those shown in Table 1), which constitute parallel mutations, are shown in italics.

Omicron (PANGO Lineage)	Detected Mutations that Define Omicron Lineages at Each Gene									
	*ORF1a*	*ORF1b*	*S*	*ORF3a*	*E*	*M*	*ORF6*	*ORF7b*	*ORF8*	*N*
BA.1	Aa: K856R L2084I A2710T T3255I P3395H I3758V Del: S2083 3674-3676	Aa: *P314L* I1566V	Aa: A67V *T95I G142D* L212I G339D S371L S373P S375F *K417N* N440K G446S S477N *T478K* E484A Q493R G496S *Q498R N501Y* Y505H T547K *D614G H655Y* N679K *P681H* N764K D796Y N856K Q954H N969K L981F Del: 69–70 143–145 211 Ins: 214EPE		Aa: T9I	Aa: D3G Q19E A63T			Aa: *S84L*	Aa: P13L *R203K G204R* *Del*: 31–33
BA.2	Aa: S135R T842I G1307S L3027F T3090I L3201F T3255I P3395H Del: *3675–3677*	Aa: *P314L* R1315C I1566V T2163I	Aa: T19I L24S *G142D* V213G G339D S371F S373P S375F T376A D405N R408S *K417N* N440K S477N *T478K* E484A Q493R *Q498R N501Y* Y505H *D614G H655Y* N679K *P681H* N764K D796Y Q954H N969K Del: 25–27	Aa: T223I	Aa: T9I	Aa: Q19E A63T	Aa: D61L		Aa: *S84L*	Aa: P13L *R203K G204R* S413R Del: 31–33
BA.3	Aa: S153R G1307S T3090I T3255I P3395H A3657V *Del*: *3675–3677*	Aa: *P314L* I1566V	Aa: A67V *T95I G142D* L212I G339D S371F S373P S375F D405N *K417N* N440K G446S S477N *T478K* E484A Q493R *Q498R N501Y* Y505H *D614G H655Y* N679K *P681H* N764K D796Y Q954H N969K Del: 69–70 143–145 211	Aa: T223I	Aa: T9I	Aa: Q19E A63T			Aa: *S84L*	Aa: P13L *R203K G204R* S413R Del: 31–33
BA.4	Aa: S135R T842I G1307S L3027F T3090I T3255I P3395H Del: 141–143 3675–3677 Nt: G12160A	Aa: *P314L* R1315C I1566V T2163I	Aa: T19I L24S *G142D* V213G G339D S371F S373P S375F T376A D405N R408S *K417N* N440K G446S *L452R* S477N *T478K* E484A F486V *Q498R N501Y* Y505H T547K *D614G H655Y* N679K *P681H* N764K D796Y Q954H N969K Del: 25–27 69–70	Aa: T223I	Aa: T9I	Aa: Q19E A63T	Aa: D61L	Aa: L11F		Aa: P13L P151S *R203K G204R* S413R Del: 31–33
BA.5	Aa: S135R T842I G1307S L1507F L3027F T3090I T3255I P3395H Del: *3675–3677* Nt: G12160A	Aa: *P314L* R1315C I1566V T2163I	Same mutations as BA.4	Aa: T223I	Aa: T9I	Aa: D3N Q19E A63T				Aa: P13L *R203K G204R* S413R Del: 31–33

**Table 3:** Parallel mutations observed among variants of concern. The table indicates the mutations that are observed in different variants of concern, with their main corresponding consequences when documented (references).

Gene	Mutation (s)	Consequences	References
*ORF1a*	Del: 3675–3677	Deletion located in the protein nsp6, which is important for the synthesis of RNA. This deletion removes amino acids from a transmembrane loop A very similar deletion was observed in the VOC Omicron BA.1 (deletion 3674–3676), which was associated with a favoring effect about increasing mutability	[42,43]
*ORF1b*	P314L	This mutation is in linkage disequilibrium with the D614G mutation of the *S* gene	[44]
*S*	E484K	This mutation induces escape to monoclonal antibodies and reduces the neutralizing capacity of convalescent and post-vaccination polyclonal sera	[45]
	Q498R	This mutation increases the affinity between SARS-CoV-2 RBD and human ACE2	[46]
	N501Y	This mutation increases the affinity between SARS-CoV-2 RBD and human ACE2	[47]
	D614G	This mutation increases infectivity by changing the formation of the spike protein to a competent state for binding with ACE2	[48]
	P681H	This mutation confers resistance to type I interferons and reduces dependence on endosomal cathepsins favoring cell entry	[49]
	K417N	This mutation reduces the activity of human and commercial antibodies but also reduces the affinity of the spike protein with human ACE2	[50]
	H655Y	This mutation increases the fusogenicity with human cell membrane by using cathepsin-mediated entry and reduces the entry using the serine transmembrane protease 2	[51,52]
	T95I	-	-
	G142D	Mutation associated with immune evasion, back mutations, and increased viral load	[53]
	L452R	This mutation increases the infectivity and fusogenicity and promotes viral replication	[54]
	T478K	This mutation increases the electrostatic potential of the spike protein at human ACE2 binding and can also play a role in immune escape	[55]
*ORF8*	S84L	-	-
*N*	R203K, G204R	These mutations increase infectivity, fitness and virulence	[56]

**Table 4:** Main detected recombination events in SARS-CoV-2 as a function of the variants involved. The table shows the main published recombination events (each line in the second column) involving VOCs and non-VOCs (first column). Note that recombination between some variants was not detected so far (i.e., between Alpha and Delta). The breakpoint interval is shown in nucleotides.

Variants Involved	Breakpoint Interval/Gene or Genes Involved	Additional Information	References
Alpha (VOC) and Epsilon (VOI)	Inside the genes S, N, and ORF8 [88]	-	[88]
Delta (VOC) and Omicron (VOC)	19220–21618/ORF1ab and S [89] 22034–22194/S [89,90,91] 22035–22577/S [84] 22204–22578/S [92] 22218–22586/S [89] 25469–25584/ORF3a [90,91]	Recombinant named “Deltacron” or “Deltamicron”. The concern about this chimera lies in the combination of the high transmissibility of Omicron and the virulence and severe disease caused by Delta [90,93]	[84,89,90,91,92,93,94,95,96,97,98]
Alpha (VOC) and B.1.160	17109–18877/ORF1ab [90,91] 25710–27972/ORF3a, E, M, ORF6, ORF7a, ORF7b [90,91]	Recombination occurred among parents of these variants	[90,91,99]
Various lineages and Alpha (VOC)	21255–21764/ORF1ab, S [83] 6528–6954/ORF1ab [83] 24914–28651/S, ORF3a, E, M, ORF6, ORF7a, ORF7b, ORF8, N [83] 21575–23063/S [83] 11396–21991/ORF1ab, S [83] Window nt 3267–5388 gene ORF1ab [83] 12534–21765/ORF1ab, S [83] 26801–27972/M, ORF6, ORF7a, ORF7b, ORF8 [83] 6954–10870/ORF1ab [83] 22775–22778/S [100]	-	[83,100]
Various lineages and Omicron (VOC)	13296–15240/ORF1ab [101] 20055–21618/ORF1ab, S [102]	-	[101,102]
Omicron parents	21593–23118/S [103]	-	[98,103]
Not involving VOCs	Inside the gene S [104] 22775–22778/S [105] 19408–19411/ORF1ab [105]	-	[76,104,105,106,107,108,109]

**Table 5:** The rate of molecular evolution of SARS-CoV-2 identified in diverse studies. The rates of evolution are shown in substitutions per site and per year and include the statistical confidence in terms of confidence interval (CI), highest posterior density interval (HDPI), or Bayesian confidence interval (BCI) at 95% significance of the estimation.

Rate of Evolution (Substitutions/Site/Year)	Statistical Confidence (95%)	References
7.31 × 10^−4^	5.95 × 10^−4^–8.68 × 10^−4^ (CI)	[11,110]
1.1 × 10^−3^	7.03 × 10^−4^–1.5 × 10^−3^ (CI)	[111]
8 × 10^−4^	–	[112]
1 × 10^−4^–1.4 × 10^−3^	–	[113]
7.8 × 10^−4^	1.1 × 10^−4^–1.5 × 10^−3^ (HPDI)	[114]
9.9 × 10^−4^	6.29 × 10^−4^–1.35 × 10^−3^ (BCI)	[81]
7.9 × 10^−4^	6.64 × 10^−4^–9.27 × 10^−4^ (HPDI)	[115]
3.547 × 10^−4^	1.112 × 10^−4^–5.969 × 10^−4^ (CI)	[116]
6.5 × 10^−3^	4.9 × 10^−3^–8.0 × 10^−3^ (HPDI)	[117]
